# Identification of Substances Produced by *Cercospora brachiata* in Absence of Light and Evaluation of Antibacterial Activity

**DOI:** 10.3390/jof7090680

**Published:** 2021-08-24

**Authors:** John K. R. P. Felisbino, Bruno S. Vieira, Alberto de Oliveira, Neiliane A. da Silva, Carlos H. G. Martins, Mariana B. Santiago, Rodrigo A. A. Munoz, Luís C. S. Cunha, Raquel M. F. Sousa

**Affiliations:** 1 Instituto de Química, Universidade Federal de Uberlândia, Uberlândia 38400-902, Brazil; johnkenedy7@hotmail.com (J.K.R.P.F.); alberto@ufu.br (A.d.O.); munoz@ufu.br (R.A.A.M.); 2 Instituto de Ciências Agrárias, Universidade Federal de Uberlândia, Monte Carmelo 38500-000, Brazil; brunovieira@ufu.br (B.S.V.); nena.a@hotmail.com (N.A.d.S.); 3 Instituto de Ciências Biomédicas, Universidade Federal de Uberlândia, Uberlândia 38400-902, Brazil; carlos.martins2@ufu.br (C.H.G.M.); mari.brentini@hotmail.com (M.B.S.); 4 Departamento de Química, Instituto Federal do Triangulo Mineiro, Uberaba 38064-100, Brazil; luisscunha@gmail.com

**Keywords:** *Cercospora brachiata*, secondary metabolites, antibacterial activity, fungus, oral bacteria

## Abstract

*Cercospora brachiata* is a phytopathogenic fungus. To know more about the metabolites produced by this fungus, the objective of this work was to identify, isolate and characterize substances present in extracts of the growth broth and mycelium, using gas chromatography with mass spectrometry (GC-MS) and nuclear magnetic resonance (NMR). It was also objective to evaluate the antibacterial activity of the extracts. Among the compounds identified, fatty acids, esters, and steroids can be highlighted. The main compounds identified are 9-hexadecenoic, hexadecenoic, oleic, octadecanoic, lauric, myristic, palmitic, doceno-13-enoic, stearic, linoleic, and nonadecanoic acids present in almost all extracts. For the antibacterial activity, the broth microdilution method was used. The ethyl acetate extract of the mycelium presented inhibitory concentrations (MICs) against the bacterium *Actinomyces naeslundii* (100 μg mL^−1^) and *Streptococcus sanguinis* (200 μg mL^−1^). Finally, two steroids were isolated and identified in the hexane extract of mycelium: ergosta-6,22-dien-3β,5α,8α-triol and brassicasterol.

## 1. Introduction

Fungi can be classified according to the type of host. Fungi that use host plants are called endophytic plants which do not cause plant diseases, while phytopathogens cause many types of diseases in theirs hosts [1], which is the case of some *Cercospora* species [2].

Phytopathogenic fungi can produce toxic substances. The toxins, therefore, are secondary metabolites produced by fungi, usually of low molecular mass (<1000 Da), that may be related to the infection process in their hosts [3].

Some fungi are also harmful to plants for being necrotrophic causing plant cells death during plant tissue colonization. One of the genera of necrothophic fungi is *Cercospora,* which produces toxins such as cercosporin involved in the pathogenicity of these fungi, that can be activated by light [4,5], causing lesions in leaves, stems, and fruits. One of the plants parasitized by one species of *Cercospora,* known as *Cercospora coffeicola,* is coffee, whose productivity can be reduced by up to 30% for this pathogen [6,7].

Fungi may also produce compounds capable of preventing the growth of bacteria. This activity is associated with the fact that fungi produce secondary metabolites capable of inhibiting the action of bacteria and even of other fungi, as in the case of extracts from cultures of *Cercospora* spp. isolates [8]. Bacteria used to test the antibacterial activity herein reported belonging to gram-positive and gram-negative types of the oral cavity. For Kumar (2013), there are more than 1000 species of bacteria in the oral cavity of a human being, and these bacteria are displaced to other parts of the body causing other diseases such as inflammation of the prostate, asthma, pneumonia, endocarditis, diabetes, etc. [9]. Thus, it is necessary to investigate and use new compounds that are capable of inhibiting the growth of these bacteria.

Thus, the objective of this work was to identify the substances present in extracts of the growth broth and mycelium extract of the fungus *Cercospora brachiata,* a foliar pathogen of *Amaranthus viridis*, and also to test the antibacterial activity of these extracts against aerobic and anaerobic bacteria of the human oral cavity.

## 2. Materials and Methods

### 2.1. Chemicals and Reagents

The following standard analytical solvents were used: hexane (Neon^®^, São Paulo, SP, Brazil), ethyl acetate (Panreac ApliChem ITW Companies^®^, Darmstadt, Germany), ethanol (Synth^®^, Diadema, Brazil), dichloromethane (Vetec Fine Chemical^®^, Rio de Janeiro, RJ, Brazil), methanol (Sigma-Aldrich^®^, San Luis, Missouri, EUA), and deuterated chloroform (Sigma-Aldrich^®^).

### 2.2. Instrumentation

The composition of extracts and isolated compounds were identified by a gas chromatograph coupled to a mass spectrometer (Shimadzu, QP2010) using a DB-5 capillary column (J&W, 30 m × 0.25 mm × 0.25 m). The conditions used were: helium as a carrier gas with a constant flow of 1.0 mL min^−1^; injector temperature of 220 °C, split 1:20; the temperature of the oven increased from 60 to 246 °C at 3 °C min^−1^ and maintained for 24 min at 246 °C; the ionizing potential of 70 eV; the range of *m*/*z* from 40 to 650. The identification of the compounds by this technique was based on the arithmetic index (AI) calculated and compared to that of Adams (2007) [10]. The similarity index (IS) obtained by the software (LabSolution-GCMS Solution) was also used by comparison with spectra present in the Nist27 libraries, Nist147, Wiley7, Wiley229, and Shim2205. The NMR analyses of ^1^H and ^13^C were carried out using a Bruker (AscendTM 400 Avance III HD model) equipment with 9.4 Tesla and 400 MHz.

### 2.3. Fungal Culture

Isolation and identification of the fungus *C. brachiata* were carried out at the Institute of Agricultural Sciences of the Federal University of Uberlândia (Monte Carmelo, MG-Brazil) by Dr. Bruno S. Vieira and collaborators [11], who reported this fungus for the first time in Brazil. The fungus was isolated from infected leaves of *Amaranthus viridis* L. (Caruru, voucher specimen VIC 47,138 deposited in the Herbarium at the Federal University of Viçosa, MG-Brazil). The fungus was grown in Petri dishes containing the potato-dextrose-agar medium (PDA) at 25 °C for 7 days. Mycelium discs (Ø = 10 mm) from the periphery of actively growing cultures were aseptically transferred to erlenmeyer containing 250 mL of the modified Jenkins–Prior liquid culture medium [12]. The erlenmeyers flasks were transferred to an orbital shaker at 150 rpm in the dark at 25 °C for 21 days. The cultures obtained were filtered using Buchner’s vacuum funnel to separate the mycelium from the growth broth [13].

### 2.4. Isolation and Purification of Compounds

Extract of growth broth from 5 L of *C. brachiata* was prepared by liquid-liquid partition using ethyl acetate (5 × 200 mL) at pH 5. After removal of solvent, 0.505 g of extract was obtained.

Extract of mycelia from 174 g of *C. brachiata* was prepared by maceration using methanol (600 mL) for 5 days at room temperature [14]. After removal of solvent, 100 mL of distilled water was added to the crude extract in a decantation flask and the liquid-liquid fraction was made using hexane (3 × 200 mL) and ethyl acetate (3 × 200 mL). After removal of solvent, 1.33 g of extract in hexane and 0.122 g of extract in ethyl acetate were obtained.

The hexane fraction of the mycelium was used for chromatography. Silica 60 G of 40–270 mesh was used as the stationary phase and ethyl acetate/hexane as mobile gradient phase, starting at a ratio of 1:4. From the first column, 24 fractions were obtained, of which fraction 8 was analyzed in NMR, and ergosta-6,22-dien-3β,5α,8α-triol was identified. The fractions 3 to 7 were pooled to make the second column. From this column, three fractions were obtained, and the compounds from fraction 1 were separated in the third column. From this column, four fractions were obtained, and fraction 3 was analyzed in NMR, and brassicasterol was identified. NMR spectra are presented as supplementary files (Figures S1–S7).

### 2.5. Antibacterial Activity

Biological assays to verify antimicrobial activity were performed by determining MIC (minimum inhibition concentration). The oral bacteria were obtained from the American Type Culture Collection (ATCC, Rockville, MD, USA). The following aerobic bacteria were used: *Streptococcus mutans* (ATCC 25175), *S. mitis* (ATCC 49456), *S. sanguinis* (ATCC 10556), *Aggregatibacter actinomycetemcomitans* (ATCC 43717). The following anaerobic bacteria were used: *Porphyromonas gingivalis* (ATCC 33277), *Fusobacterium nucleatum* (ATCC 25586) and *Actinomyces naeslundii* (ATCC 19039). The analysis of the antibacterial activity was performed in microplates containing 96 wells. The inoculum, the broth, and the sample were added to each well, yielding a final volume of 100 μL for aerobic bacteria and 200 μL for anaerobic bacteria. The volume of inoculum added in the microplate wells was 20 μL for the aerobic bacteria tests and 40 μL for the anaerobic bacteria tests. The concentrations of the samples tested against aerobic and anaerobic bacteria were from 0.195 μg mL^−1^ to 400 μg mL^−1^. Chlorhexidine (0.115 to 59.0 μg mL^−1^) was used as positive control for aerobic and anaerobic bacteria. To control the technique, metronidazole (0.0115 to 5.9 μg mL^−1^) was used as control for anaerobic bacteria against *Bacteroides fragilis* and *B. thetaiotaomicron*. Dimethylsulfoxide (DMSO) (5.0 to 1.0%, *v*/*v* concentration) was used as the solvent control. Sterility controls of the positive control, culture medium, and samples were also performed.

For aerobic bacteria, the microplates were incubated in microaerophilia by the flame/candle system at 37 °C for 24 h. After the incubation period, 30.0 μL of the resazurin indicator (0.02% *m*/*v*) was added to each well [15]. For anaerobic bacteria were incubated for 72 h in an anaerobic chamber (atmosphere containing: 5–10% H_2_, 10% CO_2_, 80–85% N_2_) at 36 °C and revealed with the same indicator [16].

## 3. Results and Discussion

### 3.1. Compounds Identified by GC-MS

Some compounds identified by GC-MS can be highlighted because they are common among the extracts of the analyzed fungi, such as long-chain fatty acids (9-hexadecenoic, hexadecanoic, oleic, octadecanoic, lauric, myristic, palmitic, docos-13-enoic, stearic, linoleic, and nonadecanoic acids) [17,18].

Fatty acids play important cellular roles in biological systems. This is because these molecules make up the constitution of the cell walls, regulate the activity of enzymes and inflammatory processes [19]. Fatty acids are long-chain carboxylic acids (hydrocarbons) and are the components of many lipids, including glycerides. As they are one of the building blocks used in the production of complex lipids, they have considerable medicinal and nutritional value. For example, gamma linolenic acid (GLA, an omega-6 fatty acid) in the form of evening primrose oil (EPO) has been used to treat rheumatoid arthritis, multiple sclerosis, schizophrenia, and premenstrual syndrome. Eicosapentaenoic acid acetic acid (EPA) and docosahexaenoic acid (DHA, both omega-3 fatty acids) have physiological effects in areas such as heart, circulation, inflammation, and cancer [20].

According to Blacklock et al. [21], there is a diversity in the synthesis of fungal fatty acids, and their identification by GC-MS can be facilitated when they are esterified, for example, methyl hexadecanoate, (Z) -9-octadecenoate, methyl octadecenoate, methyl palmitate, methyl oleate, and methyl linoleate esters were identified in fungal extracts.

Other compounds identified were steroids, which are substances found in almost every living organism, whether in plants, fungi, bacteria, or in human beings. According to Dewick [22], steroids are modified triterpenoids and their biosynthesis is the route of mevalonic acid (MVA), which is formed by three molecules of acetyl-CoA.

**Figure 1:**
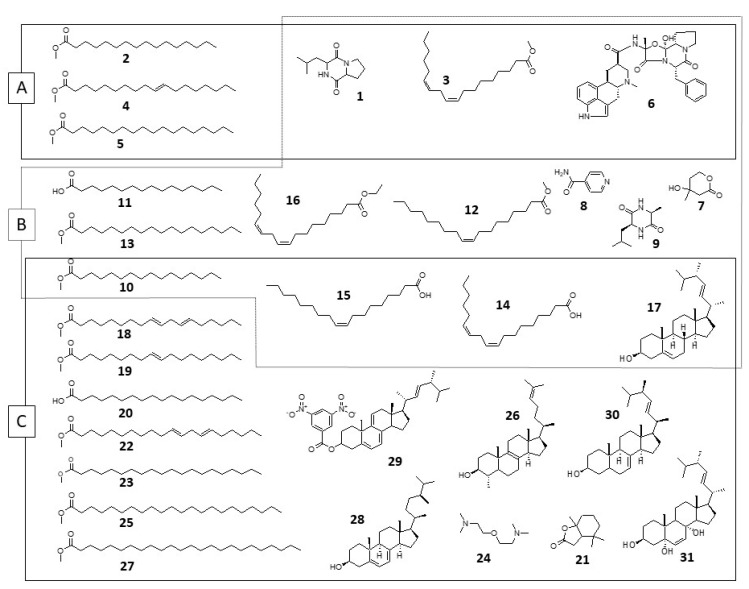
Structures of the compounds identified in *C. brachiata* extracts. (**A**): ethyl acetate from the growth broth; (**B**): ethyl acetate from mycelium; (**C**): hexane from mycelium.

Table 1 shows the compounds identified by GC-MS in the ethyl acetate extract of the fungus growth broth and the extracts in hexane and ethyl acetate from the mycelium of the fungus. The structures of the identified compounds are shown in Figure 1.

### 3.2. Isolated Compounds

The hexane fraction was fractionated through column chromatography. Fraction 8 from the first column and fraction 3 from the third column were analyzed by NMR and the compounds ergosta-6,22-dien-3β,5α,8α-triol (31), and brassicasterol (17) were characterized, respectively (Figure S1).

Ergosta-6,22-dien-3β,5α,8α-triol (31) from fraction 8 of first column 3 was analyzed by the ^13^C-NMR and 28 carbons were found in the spectrum, which matched the possible structures of the Ergosta-like molecules identified by GC-MS at retention times 75.8 to 93.9 min, with respective similarity indexes of 60 and 80, shown in Table 1. Compound 31 was not identified by GC-MS, probably because the presence of three hydroxyl groups results in a compound not volatile enough to be analyzed in the conditions used in this analysis.

Of the 28 signals observed in the ^13^C-NMR spectrum (Figure S6) of compound 31, 3 signals were in the region of carbinolic carbons, δ 67.1 (C-3), 82.7 (C-5) and 79.9 ppm (C-8); 4 signals were in the carbons methynic group at δ 136.0 (C-6), 131.3 (C-7), 132.8 (C-22), and 135.7 ppm (C-23).

From DEPT-135 (Figure S7) it was possible to verify the absence of carbons 10 and 13 which are quaternary, with displacements in δ 37.5 and 45.1 ppm, respectively. It was also verified that the carbons 5 and 8 are not quaternary, but they do not present hydrogens, therefore, do not appear in DEPT-135. Additionally, the presence of 6 carbons from methylic group in δ 13.4 (C-18), 18.7 (C-19), 18.1 (C-21), 20.5 (C- 26), 20.2 (C-27), and 21.4 ppm (C-28).

The DEPT-135 also allowed the identification of methylene carbons, with displacements in δ 35.2 (C-1), 39.8 (C-2), 37.4 (C-4) 21.2 (C-11), 30.6 (C-12), 23.9 (C-15), and 29.2 ppm (C-16), and the methyl carbons at 52.2 (C-9), 56.7 (C-14), 33.6 (C-17), 43.3 (C-20), 40.2 (C-24), and 51.6 ppm (C-25). The data obtained by the ^13^C-NMR were compared with data from Rivera, Benavides, and Rios [23] (Table 2), which confirmed the structure of Ergosta-6,22-dien-3β,5α,8α-triol (31).

The ^1^H-NMR spectrum (Figure S5) shows the signals of the methynic hydrogens H-6 (δ: 6.5, d, 1H, *J*: 8.3 Hz), H-7 (δ: 6.3, d, 1H, *J* = 8.3 Hz), H-22, and H-23 (δ: 5.1–5.2, m, 1H).

Brassicasterol (17) from fraction 3 of third column 3 was analyzes by the ^13^C-NMR and 28 carbons were found in the spectrum that matched the structure identified by GC-MS, at the retention time of 88.4 minutes, with a similarity index of 75% as shown in Table 1. The compound was identified in the fraction extracted in ethyl acetate from the mycelium, however, it was isolated from the fraction in hexane, indicating that not all compounds were extracted by the liquid–liquid partition. The GC-MS data were compared with the NMR data.

Of the 28 signals observed in the ^13^C-NMR spectrum (Figure S3), the following are highlighted: the signal with a shift in δ 72.2 ppm referring to carbinolic carbon (C-3); 4 signals referred to alkene: 141.1 ppm to the quaternary carbon (C-5), three referring to the methynic carbons in δ 122.1 (C-6), 132.1 (C-22), and 136.2 ppm (C-23).

With DEPT-135 (Figure S4) it was possible to verify the absence of carbons 10 and 13 which are quaternary, with displacements in δ 36.9 and 42.6 ppm respectively. The presence of 6 carbons methylic in δ 12.4 (C-18), 21.4 (C-19), 17.9 (C-21), 19.9 (C- 26), 19.7 (C-27), and 20.3 ppm (C-28).

It was also possible with DEPT-135 the identification of methylene carbons, with displacements in δ 31.9 (C-1), 37.6 (C-2), 42.5 (C-4), 29.9 (C-7), 21.3 (C-11), 40.0 (C-12), 28.8 (C-15), and 24.6 ppm (C-16). Additionally, methynic carbons in δ 32.2 (C-8), 50.5 (C-9), 56.3 (C-14), 57.2 (C-17), 40.5 (C-20), 43.1 (C-24), and 33.4 ppm (C-25). The data obtained by ^13^C-NMR were compared with data from Sun et al. [24] (Table 3), which confirmed the structure of the steroid Brassicasterol (17).

The ^1^H-NMR spectrum (Figure S2) shows the signal of the methynic hydrogen of carbon H-6 (δ: 5.33–5.35, m, 1H), H-22, and H-23 (δ: 5.17–5.19, m, 1H). Other hydrogen signals in the spectrum have been identified and compared with the literature, such as methynic hydrogen of carbon H-3 (δ: 3.48–3.55, m, 1H), which is hydrogen of carbinolic carbon. There are also the methylic hydrogens H-21 (δ: 0.9, d, 3H, *J*: 6.8 Hz), H-26, and H-27 (δ: 0.94, d, 3H, *J*: 6.5 Hz).

### 3.3. Antibacterial Activity

The antibacterial activity was evaluated to test the “Minimum inhibitory concentration” (MIC), that is, the minimum concentration capable of inhibiting bacterial growth. The results are shown in Table 4. The results that are considered present good activity against bacteria are those whose minimum inhibitory concentrations are close to 100 μg mL^−1^, whereas concentrations between 200 and 500 μg mL^−1^ are considered to be moderate activity [25].

Regarding aerobic bacteria, the values found for antibacterial activity are considered moderate for the hexane extract of mycelium against *S.mitis* and *S. sanguinis*; for the ethyl acetate extract of mycelium against *S. mitis*, *S. sanguinis,* and *A. actinomycetemcomitans*. The ethyl acetate extract from the broth showed no activity within the range of the concentrations tested (0.195 to 400 μg mL^−1^).

Regarding the anaerobic bacteria, the results showed that the ethyl acetate extract of the mycelium inhibited the growth of all tested bacteria with promising activity to *A. naeslundii* (100 μg mL^−1^). The hexane extract of the mycelium and ethyl acetate extract from the broth showed moderate activity against the bacteria *P. gingivalis* and *A. naeslundii* with MIC values from 200 to 400 μg mL^−1^.

Previous work has already reported the antibacterial activity of the metabolites of the fungus *Cercospora* sp. against the bacteria *Bacillus subtilis* and *Staphylococcus aureus* [8]. However, the results were given in a zone of inhibition of halo, presenting values of 15.0 ± 0.3 and 25.0 ± 0.4 mm, respectively. This antibacterial activity observed, even in a qualitative way, corroborates with the data of this work, in which the extracts of the fungus *Cercospora* sp. inhibited the growth of different oral bacteria with concentrations between 100 and 400 μg mL^−1^. Therefore, the antibacterial activity of the metabolites of this fungus is demonstrated.

Fatty acids and steroids were one of the main classes of metabolites found in the extracts, and the results found may be related to the presence of these constituents.

The antibacterial properties of fatty acids are well known, the intensity of activity can be influenced by the length and shape of the chain size and also by the presence or absence of unsaturations. Hydrophobic and hydrophilic interactions of fatty acids with the bacterial cell membrane generate structural changes in the bacterial cell. This process can affect the energy production, inhibit enzymatic activity, impair nutrient absorption, generate degradation products (auto-oxidation and peroxidation), or promote lysis of the cell membrane contributing to growth inhibition or death of the bacterium [26].

Steroids were another class of compounds identified and isolated from the extracts of the fungus *Cercospora*. Steroids can also be considered to contribute to the antimicrobial action of extracts. Other works have already demonstrated the potential of these compounds against different Gram-negative and Gram-positive bacteria [27,28,29,30]. They have an action similar to fatty acids on cell membranes, as they adhere to the lipid layers of the membranes, favoring the passage of nutrients and cell lysis [31]. Thus, fatty acids and steroids can be the bioactive compounds responsible for the activity of the extracts.

## 4. Conclusions

The chemistry of natural products continues to be an important component in science because it is in contact with the various studies related to biota, fauna, and flora. The fungi are part of this biological system and their chemical and biological analyses bring important contributions to clarify the chemical composition of the compounds produced by these fungi and correlations of chemical profiles between genera and species can be obtained.

Among the compounds identified, fatty acids, esters, and steroids were found, which have structural functions in the cell membrane, so it is reasonable that they are found in fungus extracts.

In this work, the antibacterial activity of *Cercospora brachiata* extracts was evaluated. It was observed that the extracts from extractions with ethyl acetate presented promising values against anaerobic bacteria of the oral cavity.

## Figures and Tables

**Table 1:** Compounds identified by GC-MS of *C. brachiata* extracts.

Compounds		Sample *	TR (min)	TIC (%)	AI Observed	AI Literature **	Library	SI
1	1,4-diaza-2,5-dioxo-3-isobutylbicyclo-[4.3.0]-nonane	B/A	42.4	23.31/12.71	-	-	WILEY7	92/91
2	Hexadecanoic acid, methyl ester	A	43.2	5.88	1935	1933	NIST27	93
3	Linoleic acid, methyl ester	B/A	48.4	4.95/8.09	2101	2092	WILEY7	96/94
4	10-octadecenoic acid, methyl ester	A	48.6	8.44	2108	2110	WILEY229	95
5	Octadecanoic acid, methyl ester	A	49.5	1.53	2138	2135	WILEY7	90
6	Ergotaman-3′, 6′, 18-trione, 9,10-dihydro-12 ‘-hydroxy-2′-methyl-5′- (phenylmethyl)-,(5′, 10a)	B/A	56.1	6.21/3.18	-	-	NIST27	86/87
7	Mevalonic lactone	B	17.7	1.52	1260	-		92
8	Isonicotinamide	B	23.5	1.66	1408	1426	WILEY7	92
9	Cycloalanylleucine	B	36.2	4.61	-	-	WILEY7	92
10	Hexadecanoate, methyl ester	B/C	43.2	4.12/5.88	1935	1926	NIST27	96/96
11	Palmitic acid	B	44.4	3.12	1971	1975	NIST27	94
12	Oleic acid, methyl ester	B	48.7	4.92	2111	2103	WILEY7	95
13	Octadecanoic acid, methyl ester	B	49.5	1.14	2138	2138	WILEY229	91/96
14	Linoleic acid	B/C	49.7	2.61/38.13	2145	2132	WILEY7	91/91
15	Oleic acid	B/C	49.9	6.85/6.26	2151	2141	WILEY7	86/92
16	Linoleic acid, ethyl ester	B	50.5	0.61	2169	2163	NIST27	84
17	Brassicasterol	B/C	88.4	3.45/3.58	-	-	WILEY7	75/81
18	Octadeca-9,12-dienonoic acid, methyl ester	C	48.8	19.52	1862	-	WILEY7	9 7
19	(*E*) -9-Octadecenonoic acid, methyl ester	C	49.0	12.05	2122	2110	NIST27	96
20	Stearic acid	C	51.2	2.05	2196	2188	NIST27	92
21	Hexahydro-4,4,7a-trimethyl-2-benzofuranone	C	54.2	0.17	2303	2305	WILEY229	80
22	11,14-Eicosadienonoic acid, methyl ester	C	54.5	0.08	2313	-	WILEY7	96
23	Eicosanonoic acid, methyl ester	C	55.4	0.21	2374	2339	WILEY7	95
24	*N*, *N*, *N′*, *N′*-Tetramethyl-2,2′-oxybis (ethylamine)	C	58.8	0.30	2475	-	NIST27	92
25	Docosanonoic acid, methyl ester	C	60.7	0.04	2547	2530	NIST27	94
26	Cholesta-8,24-dien-3-ol, 4-methyl-, (3β, 4α)	C	61.9	0.06	2597	-	WILEY229	78
27	Tetracosanoic acid, methyl ester	C	66.4	0.16	2739	2731	WILEY7	95
28	Ergosta-5,7-dien-3-ol, (3β)	C	75.8	0.73	2948	-	WILEY7	60
29	(22E)-Ergosta-5,7,9(11),22-tetraen-3-yl 3,5-dinitrobenzoate	C	77.4	0.25	2980	-	NIST27	71
30	Ergosta-7,22-dien-3-ol, (3β, 22E)	C	93.9	0.57	-	-	WILEY229	80

Note: * A: ethyl acetate from the growth broth; B: ethyl acetate from mycelia; C: hexane from mycelia. ** Adams (2007) [10].

**Table 2:** ^1^H and ^13^C NMR data of ergosta-6,22-dien-3β,5α,8α-triol (31).

	Experimental Data			Literature [23]	
Carbon	^1^H NMR (400 MHz, CDCl_3_) δ (m, Integral, *J*)	^13^C NMR (100 MHz, CDCl_3_) δ	DEPT-135	^1^H NMR (400 MHz, CDCl_3_) δ (m, Integral, *J*)	^13^C NMR (100 MHz, CDCl_3_) δ
1	1.20–2.1 (m, 2H)	35.2	CH_2_	1.96 (m, 1Hα) 1.69 (m, 1Hβ)	34.7
2	1.20–2.1 (m, 2H)	39.8	CH_2_	1.99 (m, 1Hβ) 1.24 (m, 1Hα)	39.2
3		67.1	CH		66.5
4	1.20–2.1 (m, 2H)	37.4	CH_2_	2.11 e 1.92 (m, 2H)	36.9
5		82.7	C		82.2
6	6.5 (d, 1H, 8.3 Hz)	136.0	CH	6.6 (d, 1H, 8.5 Hz)	135.4
7	6.3 (d, 1H, 8.3 Hz)	131.3	CH	6.33 (d, 1H, 8.5 Hz)	130.7
8		79.9	C		79.4
9	1.20–2.1 (m, 1H)	52.2	CH	1.59 (m, 1H)	21.7
10		37.5	C		37.0
11	1.20–2.1 (m, 2H)	21.2	CH_2_	1.62 (m, 2H)	20.6
12	1.20–2.1 (m, 2H)	30.6	CH_2_	1.82 and 1.55 (m, 2H)	30.1
13		45.1	C		44.6
14	1.20–2.1 (m, 1H)	56.7	CH	1.19 (m, 1H)	56.2
15	1.20–2.1 (m, 2H)	23.9	CH_2_	1.22 (m, 2H)	23.4
16	1.20–2.1 (m, 2H)	29.2	CH_2_	1.33 (m, 2H)	28.6
17		33.6	CH		33.1
18	0.82 (s, 3H)	13.4	CH_3_	0.82 (s, 3H)	12.9
19	0.88 (s, 3H)	18.7	CH_3_	0.89 (s, 3H)	18.2
20	1.20–2.1 (m, 1H)	43.3	CH	1.82 (m, 1H)	42.8
21	0.90 (d, 3H, 6.5 Hz)	18.1	CH_3_	0.92 (d, 3H, 6.3 Hz)	17.6
22	5.1–5.2 (m, 1H)	132.8	CH	5.18 (m, 1H)	132.3
23	5.1–5.2 (m, 1H)	135.7	CH	5.22 (m, 1H)	135.2
24	1.20–2.1 (m, 1H)	40.2	CH	2.02 (m, 1H)	39.7
25	1.20–2.1 (m, 1H)	51.6	CH	1.48 (m, 1H)	51.1
26	0.80–0.84 (m, 3H)	20.5	CH_3_	0.85 (d, 3H, 6.6 Hz)	19.9
27	0.80–0.84 (m, 3H)	20.2	CH_3_	0.81 (d, 3H, 6.6 Hz)	19.6
28	1.0 (d, 3H, 6.7 Hz)	21.4	CH_3_	1.01 (d, 3H, 6.6 Hz)	20.9

**Table 3:** ^1^H and ^13^C NMR data of brassicasterol (17).

Experimental Data				Literature [24]	
Carbon	^1^H NMR (400 MHz, CDCl_3_) δ (m, Integral, *J*)	^13^C NMR (100 MHz, CDCl_3_) δ	DEPT-135	^1^H NMR (400 MHz, CDCl_3_) δ (m, Integral, *J*)	^13^C NMR (100 MHz, CDCl_3_) δ
1		31.9	CH_2_		31.8
2		37.6	CH_2_		37.4
3	3.48–3.55 (m, 1H)	72.2	CH	3.55(m, 1H)	72.0
4		42.5	CH_2_		42.4
5		141.1	C		140.9
6	5.33–5.35 (m, 1H)	122.1	CH	5.34 (m, 1H)	121.9
7		29.9	CH_2_		29.9
8		32.2	CH		32.1
9		50.5	CH		50.3
10		36.9	C		36.7
11		21.3	CH_2_		21.2
12		40.0	CH_2_		39.8
13		42.6	C		42.5
14		56.3	CH		56.2
15		28.8	CH_2_		28.7
16		24.6	CH_2_		24.4
17		57.2	CH		57.0
18		12.4	CH_3_		12.2
19		21.4	CH_3_		21.1
20		40.5	CH		40.3
21	0.9 (d, 3H, 6.8 Hz)	17.9	CH_3_	0.91 (d, 3H. 6.8 Hz)	17.8
22	5.17–5.19 (m, 1H)	132.1	CH	5.18 (m, 1H)	131.9
23	5.17–5.19 (m, 1H)	136.2	CH		136.0
24		43.1	CH		43.0
25		33.4	CH		33.3
26	0.94 (d, 3H, 6.5 Hz)	20.0	CH_3_	0.83 (d, 3H, 6.5 Hz)	19.8
27	0.94 (d, 3H, 6.5 Hz)	19.7	CH_3_	0.82 (d, 3H, 6.5 Hz)	19.6
28	0.72 (s, 3H)	20.3	CH_3_	0.69 (s, 3H)	20.1

**Table 4:** Minimum inhibitory concentration of *C. brachiata* extracts against oral bacteria.

Microorganisms		Minimum Inhibitory Concentration (MIC—µg mL^−1^)			
		A	B	C	Positive Control *
*Aerobic* *bacteria*	*S. mutans* ^1^	>400	>400	>400	0.92
	*S. mitis* ^1^	>400	400	200	3.68
	*S. sanguinis* ^1^	>400	200	400	0.92
	*A. actinomycetemcomitans* ^2^	>400	400	>400	0.46
*Anaerobic* *bacteria*	*P. gingivalis* ^2^	400	200	400	3.68
	*F. nucleatum* ^2^	>400	400	>400	1.84
	*A. naeslundii* ^1^	200	100	400	1.84

Note: A: ethyl acetate from the growth broth; B: ethyl acetate from mycelia; C: hexane from mycelia. ^1^ Gram-positive bacteria; ^2^ Gram-negative bacteria. * Positive control: chlorhexidine dihydrochloride. Bacteria to control of the technique by protocol M11-A7 CLSI [16]: *B. fragilis* (MIC = 0.7 μg mL^–1^) and *B. thetaiotaomicron* (MIC = 1.5 μg mL^–1^).

## Supplementary Materials

The following are available online at https://www.mdpi.com/article/10.3390/jof7090680/s1, Figure S1: Structure of the isolated molecules of *C. brachiata*, Figure S2: ^1^H NMR spectra (400 MHz, CDCl_3_) of Brassicasterol (17), Figure S3: ^13^C NMR spectra (100 MHz, CDCl_3_) of Brassicasterol (17), Figure S4: DEPT-135 NMR spectra (100 MHz, CDCl_3_) of Brassicasterol (17), Figure S5: ^1^H NMR spectra (400 MHz, CDCl_3_) of Ergosta-6,22-dien-3β,5α,8α-triol (31), Figure S6: ^13^C NMR spectra (100 MHz, CDCl_3_) of Ergosta-6,22-dien-3β,5α,8α-triol (31), Figure S7: DEPT-135 NMR spectra (100 MHz, CDCl_3_) of Ergosta-6,22-dien-3β,5α,8α-triol (31).
