# Supplementary material for: Identification of Substances Produced by Cercospora brachiata in Absence of Light and Evaluation of Antibacterial Activity
Source: J Fungi (Basel). 2021 Aug 24;7(9):680. doi: 10.3390/jof7090680 (PMC8469931; doi:10.3390/jof7090680)
Supplement: Supplementary file 1 [file jof-07-00680-s001.zip › jof-1343134-supplementary.pdf]

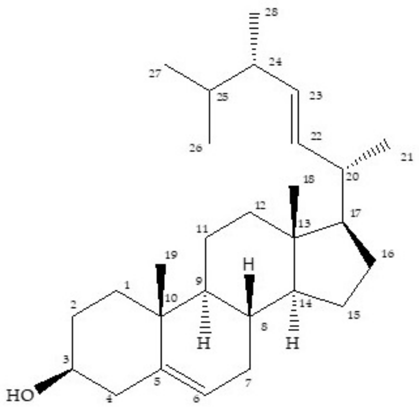

Brassicasterol (17)

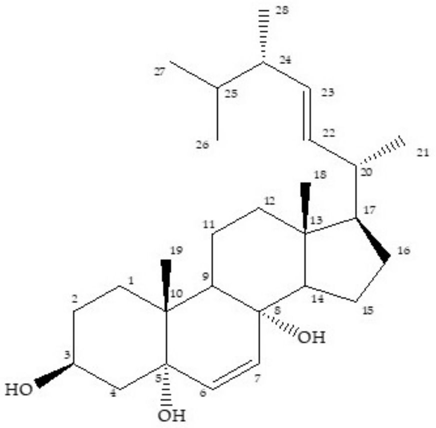

Ergosta-6,22-dien-3β,5α,8α-triol (31)

Figure S1. Structure of the isolated molecules of *C. brachiata*.

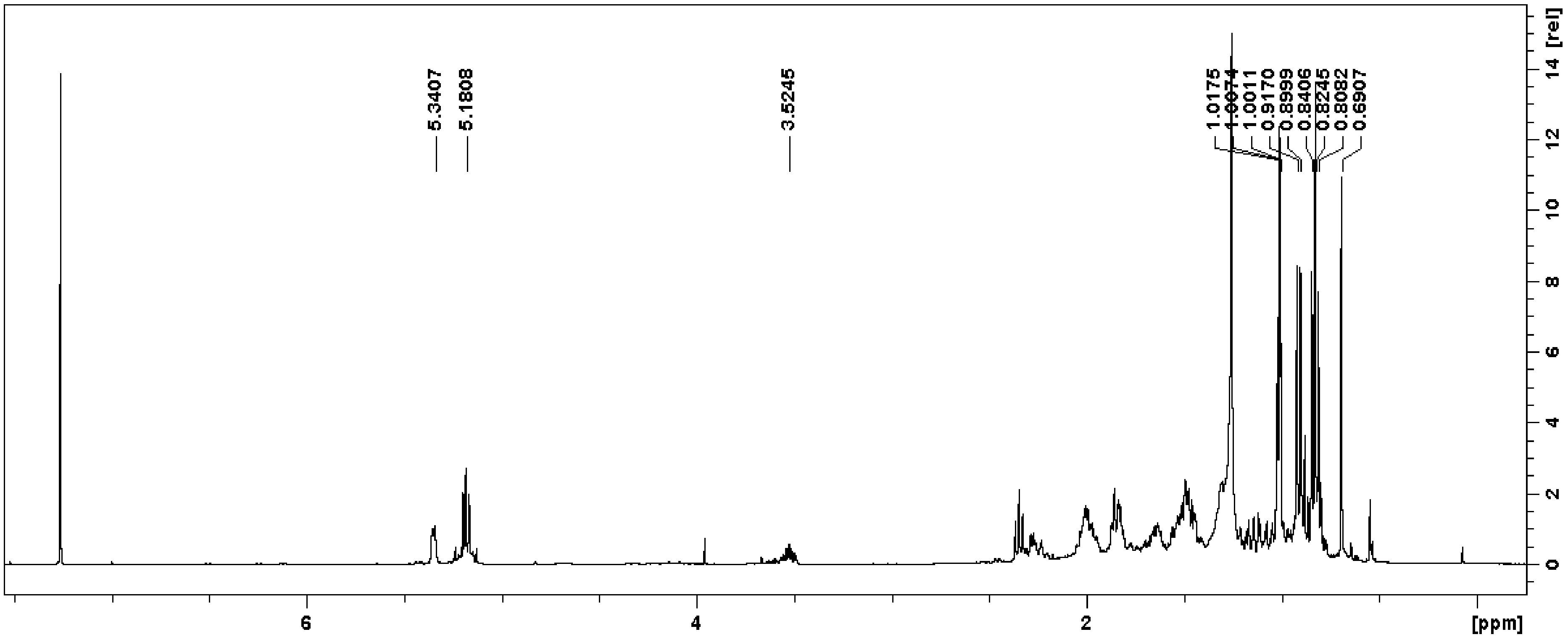

Figure S2. ^{1}H NMR spectra (400 MHz, $CDCl_3$) of Brassicasterol (17).

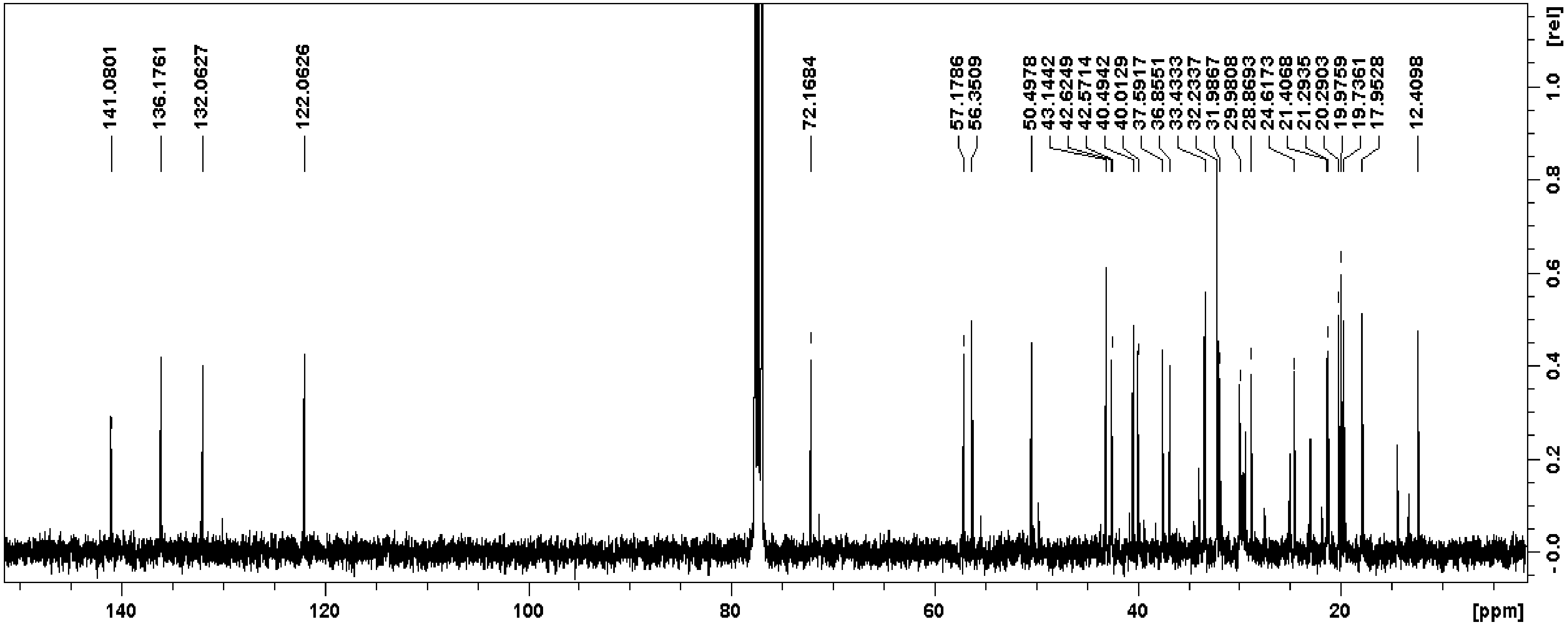

Figure S3. ^{13}C NMR spectra (100 MHz, $CDCl_3$) of Brassicasterol (17).

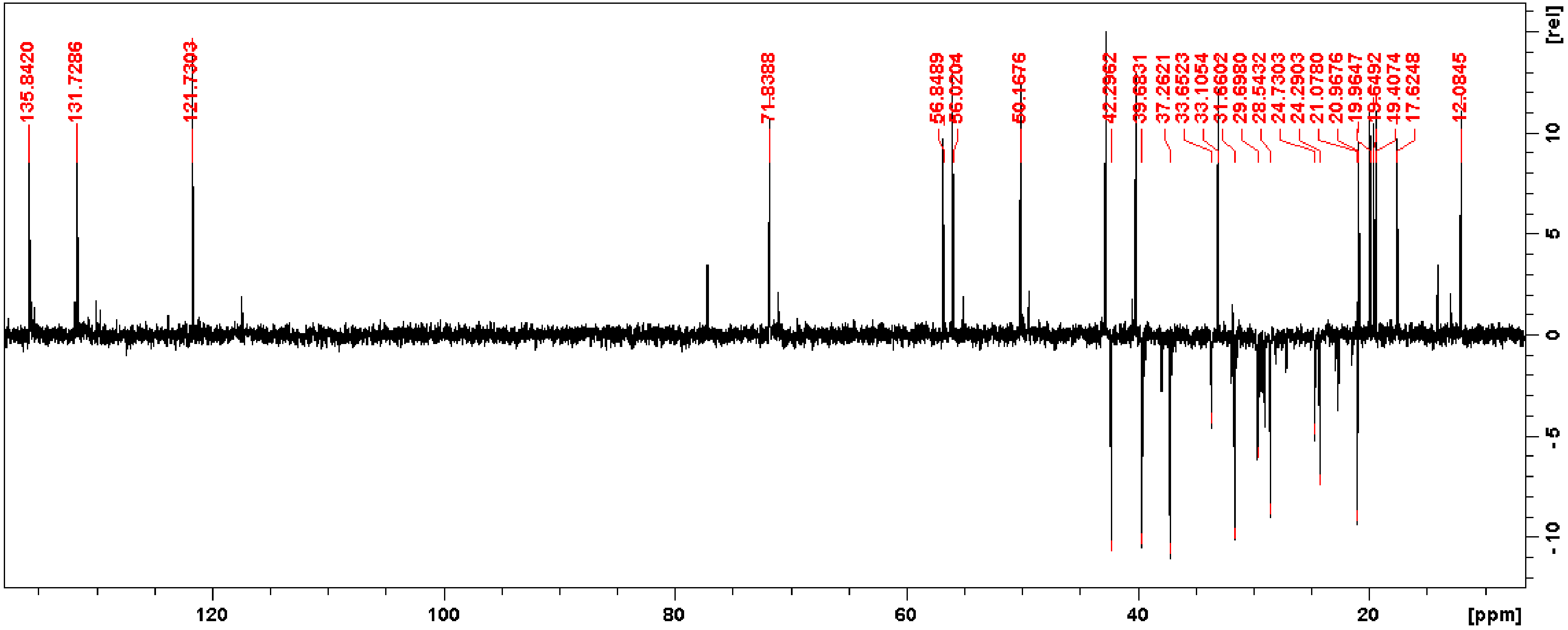

Figure S4. DEPT-135 NMR spectra (100 MHz, $CDCl_3$) of Brassicasterol (17).

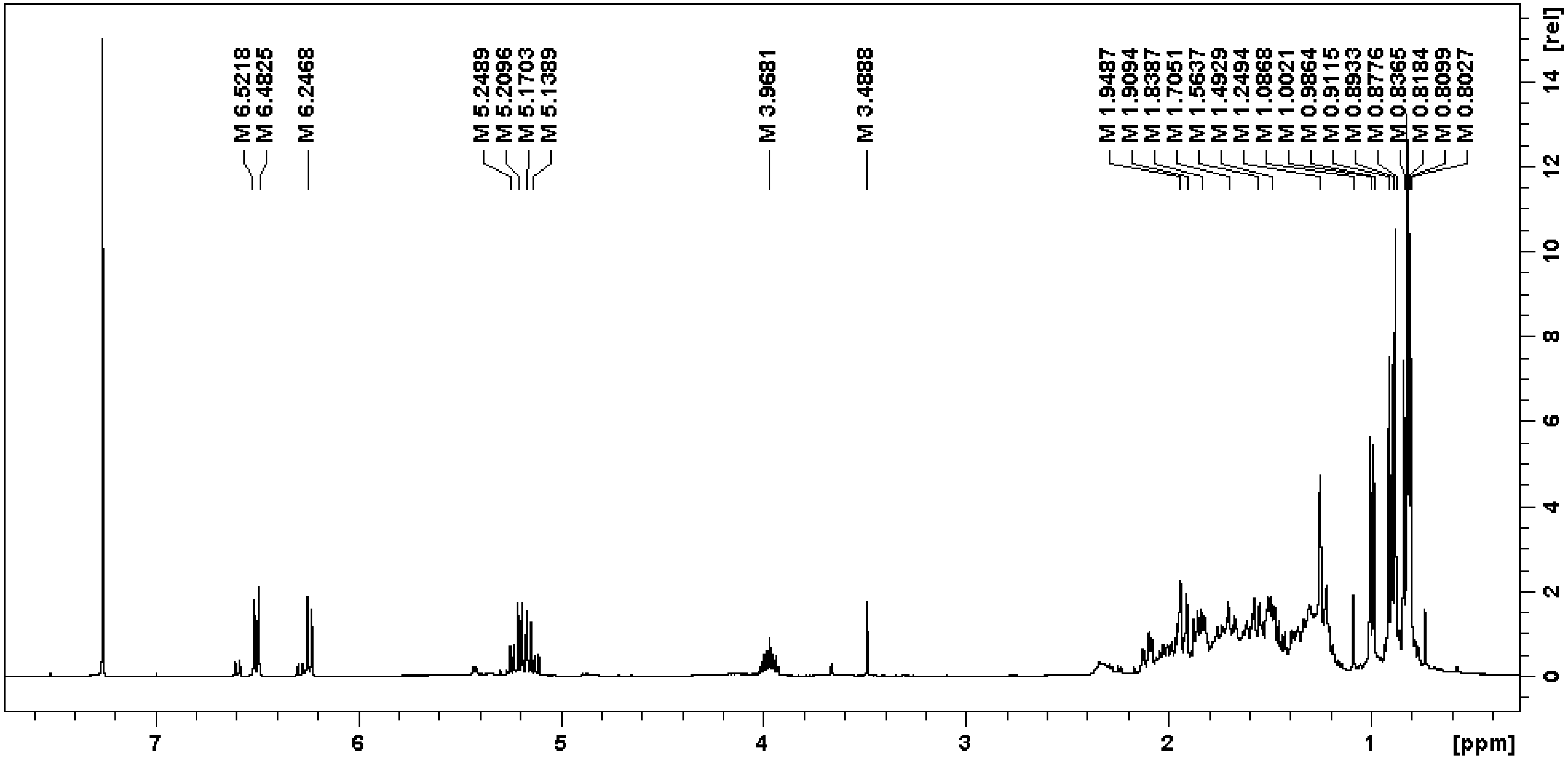

Figure S5. ^{1}H NMR spectra (400 MHz, $CDCl_3$) of Ergosta-6,22-dien-3β,5α,8α-triol (31).

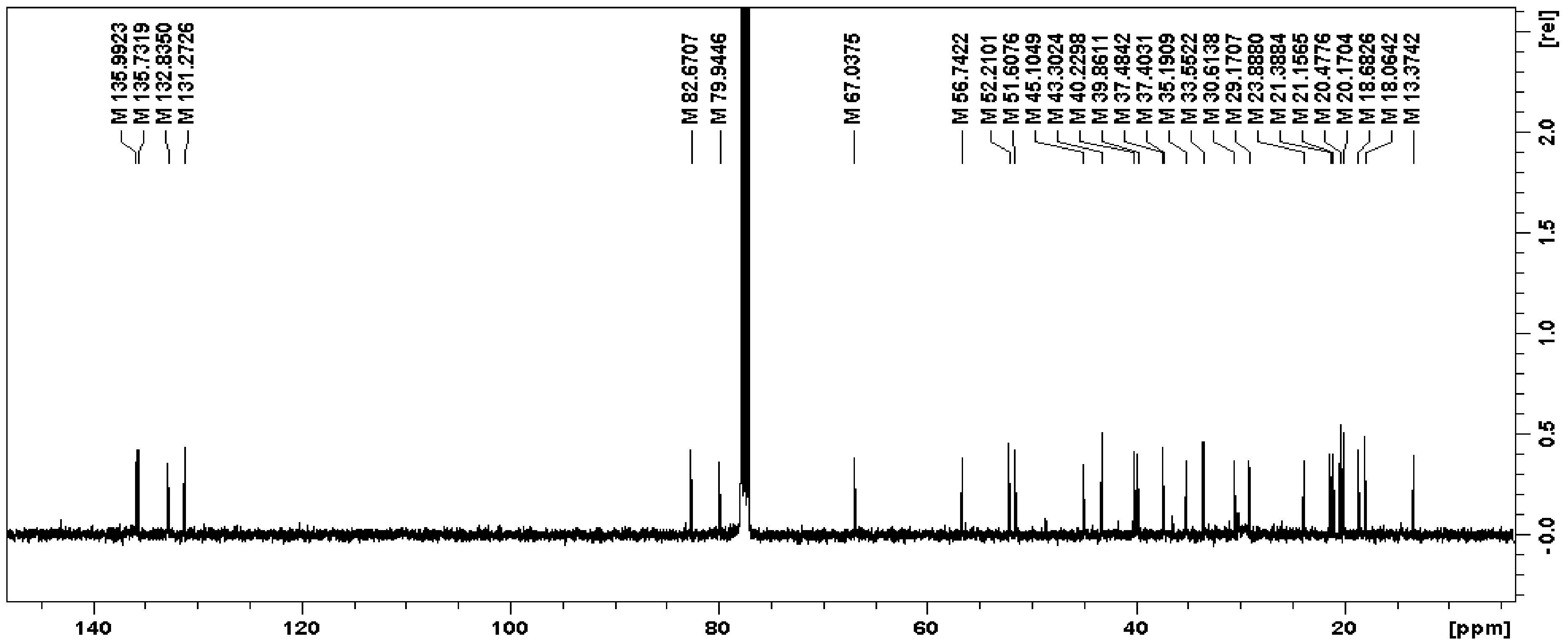

Figure S6. ^{13}C NMR spectra (100 MHz, $CDCl_3$) of Ergosta-6,22-dien-3β,5α,8α-triol (31).

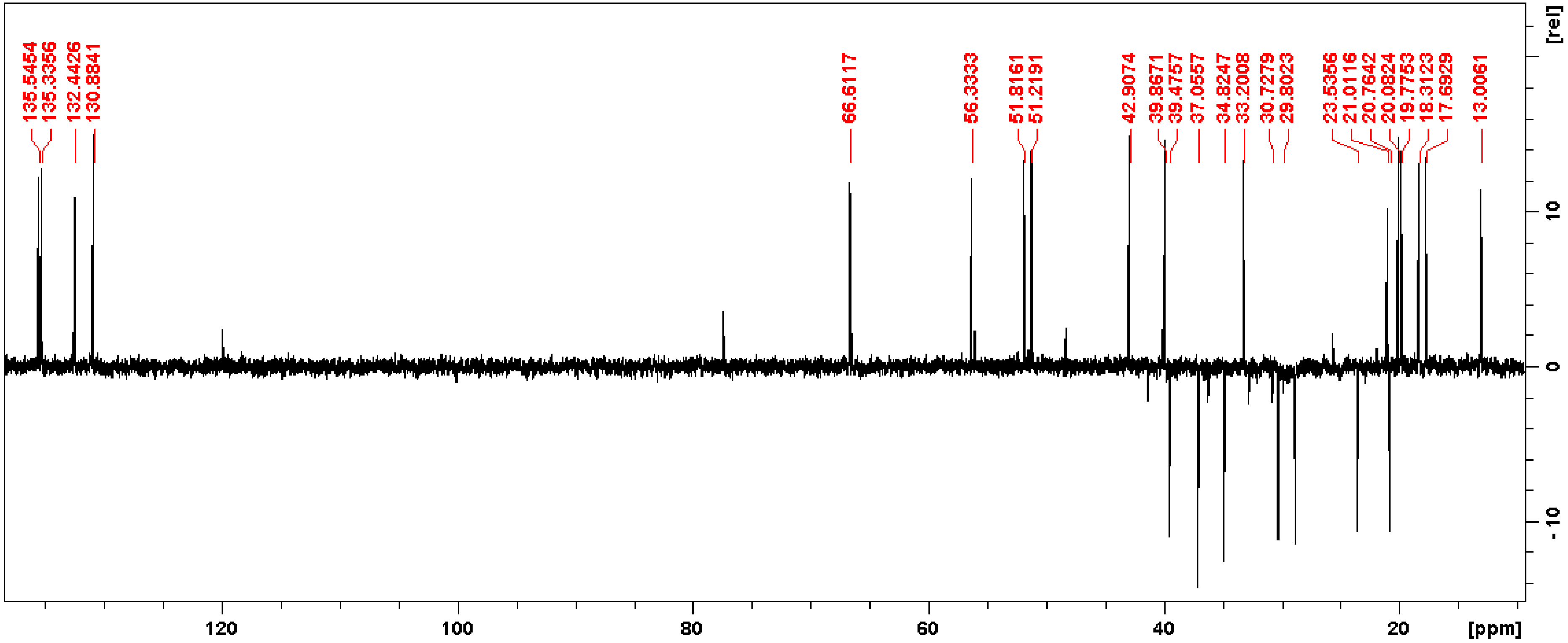

Figure S7. DEPT-135 NMR spectra (100 MHz, $CDCl_3$) of Ergosta-6,22-dien-3β,5α,8α-triol (31).
